# Simultaneous hydrolysis with lipase and fermentation of rapeseed cake for iturin A production by *Bacillus amyloliquefaciens* CX-20

**DOI:** 10.1186/s12896-019-0591-x

**Published:** 2019-12-16

**Authors:** Wenchao Chen, Xuan Li, Xuli Ma, Shouwen Chen, Yanping Kang, Minmin Yang, Fenghong Huang, Xia Wan

**Affiliations:** 1 Oil Crops Research Institute of the Chinese Academy of Agricultural Sciences, Wuhan, 430062 People’s Republic of China; 2 Key Laboratory of Biology and Genetic Improvement of Oil Crops, Ministry of Agriculture, Wuhan, 430062 People’s Republic of China; 3 Hubei Collaborative Innovation Center for Green Transformation of Bio-Resources, Environmental Microbial Technology Center of Hubei Province, College of Life Sciences, Hubei University, Wuhan, 430062 People’s Republic of China; 4 Oil Crops and Lipids Process Technology National & Local Joint Engineering Laboratory, Wuhan, 430062 People’s Republic of China; 5 Hubei Key Laboratory of Lipid Chemistry and Nutrition, Wuhan, 430062 People’s Republic of China

**Keywords:** Rapeseed cake, Rapeseed oil, Lipase, Iturin A, *Bacillus amyloliquefaciens*

## Abstract

**Background:**

Rapeseed cake (RSC), as the intermediate by-product of oil extraction from the seeds of *Brassica napus*, can be converted into rapeseed meal (RSM) by solvent extraction to remove oil. However, compared with RSM, RSC has been rarely used as a raw material for microbial fermentation, although both RSC and RSM are mainly composed of proteins, carbohydrates and minerals. In this study, we investigated the feasibility of using untreated low-cost RSC as nitrogen source to produce the valuable cyclic lipopeptide antibiotic iturin A using *Bacillus amyloliquefaciens* CX-20 in submerged fermentation. Especially, the effect of oil in RSC on iturin A production and the possibility of using lipases to improve the iturin A production were analyzed in batch fermentation.

**Results:**

The maximum production of iturin A was 0.82 g/L at the optimal initial RSC and glucose concentrations of 90 and 60 g/L, respectively. When RSC was substituted with RSM as nitrogen source based on equal protein content, the final concentration of iturin A was improved to 0.95 g/L. The production of iturin A was further increased by the addition of different lipase concentrations from 0.1 to 5 U/mL into the RSC medium for simultaneous hydrolysis and fermentation. At the optimal lipase concentration of 0.5 U/mL, the maximal production of iturin A reached 1.14 g/L, which was 38.15% higher than that without any lipase supplement. Although rapeseed oil and lipase were firstly shown to have negative effects on iturin A production, and the effect would be greater if the concentration of either was increased, their respective negative effects were reduced when used together.

**Conclusions:**

Appropriate relative concentrations of lipase and rapeseed oil were demonstrated to support optimal iturin A production. And simultaneous hydrolysis with lipase and fermentation was an effective way to produce iturin A from RSC using *B. amyloliquefaciens* CX-20.

## Background

In conventional agricultural cultivation, easy-to-use chemical pesticides and fungicides are commonly used to control plant diseases, which are a major threat to food security worldwide. However, the overuse of these chemicals has raised great concerns due to their side effects such as environmental contamination and harm to human health by the presence of chemical residues in food [1]. Biological control offers an eco-friendly and sustainable alternative and is become increasingly prevalent in modern agriculture. Among a variety of microorganisms, the genus *Bacillus* has been widely used as biological control agent due to its production of antimicrobial substances such as the cyclic lipopeptides iturin, fengycin and surfactin [2]. Iturin A, a prominent member of the iturin group, was found to suppress many plant diseases via a combination of its broad-spectrum antifungal activity and the activation of plant defense systems [3]. This makes iturin A an ideal potential biological control agent for reducing the use of artificial chemical pesticides in agriculture. In recent years, many attempts have been made to use agricultural by-products, such as rapeseed meal (RSM) [4, 5], soybean curd residue [6] and fish meal [7], to realize low-cost and large-scale production of iturin A. For example, Jin et al. [4, 5] used untreated RSM as the nitrogen source for the production of iturin A using *B. subtilis* and enhanced the production two-fold by using a two-stage glucose feeding strategy in liquid fermentation. Yao et al. [8] used *B. subtilis* to co-produce iturin A and poly-γ-glutamic acid from RSM in solid-state fermentation.

Rapeseed cake (RSC) and RSM are the major by-products in the production of rapeseed oil and are mainly composed of proteins, carbohydrates and minerals. Both are good protein resources for animal feed, with a favorable balance of essential and sulfur-containing amino acids [9]. The production process of RSM from RSC involves solvent extraction to remove oil and heating to remove the organic solvent. Although this procedure increases the protein content of RSM compared with RSC, and RSC only contains 30–40% crude protein in dry matter [10], RSC is considered more suitable for animal diets than solvent-extracted RSM by many scholars. The reasons are as follows: (1) the production technology of RSC is more environmentally friendly [11], (2) RSC has a higher metabolizable energy value due to its higher residual oil content [12], and (3) organic animal production precludes the use of oilseed meal due to the exposure to chemical solvents during the extraction [13]. However, compared with RSM [4, 5, 14–18], there are fewer reports on the use of RSC as a nutrient for microbial fermentations. In addition to obtaining more oil and profits by chemical solvent-extraction, one of the key factors is the high content of oil in RSC, which can influence the titer and productivity of some secondary metabolites [19]. Therefore, if the problem of high oil content in RSC can be solved, it is likely that RSC can be used for microbial production similarly to RSM.

Lipases, which not only hydrolyze triacylglycerides to form glycerol and fatty acids, but can also catalyze the synthesis of esters under certain conditions [20], constitute one of the most important families of industrial enzymes. Following carbohydrases and proteases, lipases are considered the third largest group of enzymes based on total sales volume [21], and are widely applied in the manufacture of foods, fine chemicals, cosmetics, pharmaceuticals, detergents, wastewater treatment, leather processing and biomedical assays [22]. However, unlike carbohydrases and proteases [16], lipases have not been widely used for the pretreatment of rapeseed by-products to improve their value for microbial production. It has been reported that RSC could be used as a valuable raw material for producing lipases and proteases due to its high content of lipids and proteins [23]. Therefore, it seems feasible to use lipases to solve the problem of high oil content in RSC.

RSM has been demonstrated as a more effective nitrogen source than two different commercial nitrogen sources for iturin A production by *Bacillus* [4]. However, to our best knowledge, there are no reports on the production of iturin A by the fermentation of RSC. In this study, we investigated the feasibility of using untreated low-cost RSC as nitrogen source to produce the valuable cyclic lipopeptide antibiotic iturin A using *B. amyloliquefaciens* CX-20 in submerged fermentation. Especially, the effect of oil in RSC on iturin A production and the possibility of using lipases to improve the iturin A production were analyzed in batch fermentation.

## Methods

### Raw material and enzyme

RSC and RSM used in the experiments were kindly supplied by the Oil Crops Research Institute of the Chinese Academy of Agricultural Sciences (Wuhan, China) and were milled in a commercial plant. The composition of RSC and RSM flour was determined and was shown in Table 1.

**Table 1:** The components of RSM and RSC

	Moisture (%)	Ash (%)	Crude Protein (%)	Crude Fat (%)	Crude fiber (%)	Neutral detergent fiber (%)	Acid detergent fiber (%)
RSM	8.3	7.3	39.4	1.6	8.8	26.6	12.8
RSC	4.7	7.0	33.5	14.4	7.1	16.6	11.7

The lipase used in this study was kindly supplied by Wuhan Sunhy Biology Co., Ltd. (China). The product is a lipase mixture used as a feed additive with a nominal activity of 10,000 U/g.

### Microorganisms and media

*B. amyloliquefaciens* CX-20 (CCTCC No. M2018794), a iturin A high- production strain, was kindly provided by professor Shouwen Chen (College of Life Sciences, Hubei University, Wuhan, China). Luria-Bertani (LB) medium (10 g tryptone, 5 g yeast extract, 10 g NaCl, and 1 L H_2_O) was used for seed cultures of *Bacillus*. The fermentation medium was composed of 60 g glucose, 1 g K_2_HPO_4_·3H_2_O, 0.5 g MgSO_4_·7H_2_O, 0.005 g MnSO_4_·H_2_O, 90 g RSC or 76.52 g RSM, and 1 L H_2_O. Flask experiments were performed in 250 mL flasks with 20 mL of medium. Due to the insolubility of RSM/RSC, 1.80 g RSC or 1.53 g RSM was weighed and placed into each 250 mL flask in advance, respectively. And then mix with distilled water containing above mentioned concentrations of inorganic salts and glucose to a final volume of 20 mL. The initial pH of the medium was adjusted to 7.0 and it was autoclaved at 121 °C for 30 min. The inoculum size was 5% (v/v). All fermentations were carried out at 28 °C under constant orbital shaking at 220 rpm.

### Simultaneous hydrolysis and fermentation

Batch submerged fermentation experiments were performed in 250 mL flasks with an initial working volume of 20 mL. The medium for submerged fermentation contained (per liter): 60 g glucose, 1 g K_2_HPO_4_·3H_2_O, 0.5 g MgSO_4_·7H_2_O, 0.005 g MnSO_4_·H_2_O, 90 g RSC or 76.52 g RSM. Due to the insolubility of RSM/RSC, 1.80 g RSC was weighed and placed into each 250 mL flask in advance, respectively. And then mix with distilled water containing above mentioned concentrations of inorganic salts and glucose to a final volume of 20 mL. Submerged fermentation was started by adding 5% (v/v) of exponentially growing cells and lipase. Submerged fermentation was conducted at 28 °C with a rotating speed of 220 rpm. The effects of lipase loading (0, 0.1, 0.5, 1, 5, 10 U/mL) and rapeseed oil concentrations (0, 6, 12 and 24 g/L) on submerged fermentation were investigated.

### Extraction and quantitation of iturin A

Iturin A was extracted according to a reported method [4, 5, 24]. Briefly, 0.3 mL of the mixed fermentation broth was added into a 2 mL glass tube containing 1.2 mL of methanol, shaken well and incubated for 60 min. The mixture was centrifuged at 12,000 rpm for 20 min, and the supernatant was filtered through a 0.22 μm pore-size hydrophobic polytetrafluoroethylene (PTFE) type disposable syringe. The iturin A concentration was quantified using a Waters 2695 HPLC system equipped with an ACQUITY UPLC BEA C18 column (1.7 μm 2.1 × 100 mm, Waters, USA). A mixture of acetonitrile and 10 mM ammonium acetate (35:65, v/v) was used as the mobile phase at a flow rate of 0.3 mL/min, and the elution was monitored at 210 nm. The concentration of iturin A was analyzed and quantified using an authentic reference standard (Sigma Chemicals, St. Louis, MO, USA). The content of iturin A was measured using triplicate samples.

### Determination of reducing sugar, free ammonium nitrogen concentrations and the viable cells

The concentrations of reducing sugar in the fermentation were determined by the DNS method using 3, 5-dinitrosalicylic acid reagent [25]. The concentration of free ammonium nitrogen (FAN) was determined using the ninhydrin colorimetric method [26]. The viable cell count during submerged fermentation was determined as follows: 0.5 mL of the sample was placed into a sterile 10 mL test tube, mixed thoroughly with 4.5 ml of sterile distilled water and shaken at 150 rpm on a vortex for 5 min at room temperature. The mixture was then serially diluted and spread onto LB agar plates. After 24 h of incubation at 28 °C, the number of colonies was counted and expressed as colony forming units (CFU).

### Determination of the iturin A stability to lipase

First, submerged fermentation were performed in 250 mL flasks with 20 mL medium, containing (per liter): 60 g glucose, 1 g K_2_HPO_4_·3H_2_O, 0.5 g MgSO_4_·7H_2_O, 0.005 g MnSO_4_·H_2_O, and 76.52 g RSM. After 72 h of fermentation, the fermentation broth was divided into two groups. Therein, one group supplemented with 5 U/mL lipase while the other without lipase addition. Then submerged fermentation was continued to be conducted at 28 °C with a rotating speed of 220 rpm. And the concentrations of iturin A at different time (0, 24, 48 and 72 h) were measured.

### Statistical analysis

All experiments were performed in triplicate and the data were processed using Origin v8.6 software (Origin Lab Corp., Northampton, MA, USA).

## Results

### Influence of initial RSC and glucose concentrations on the production of iturin A

**Fig. 1:**
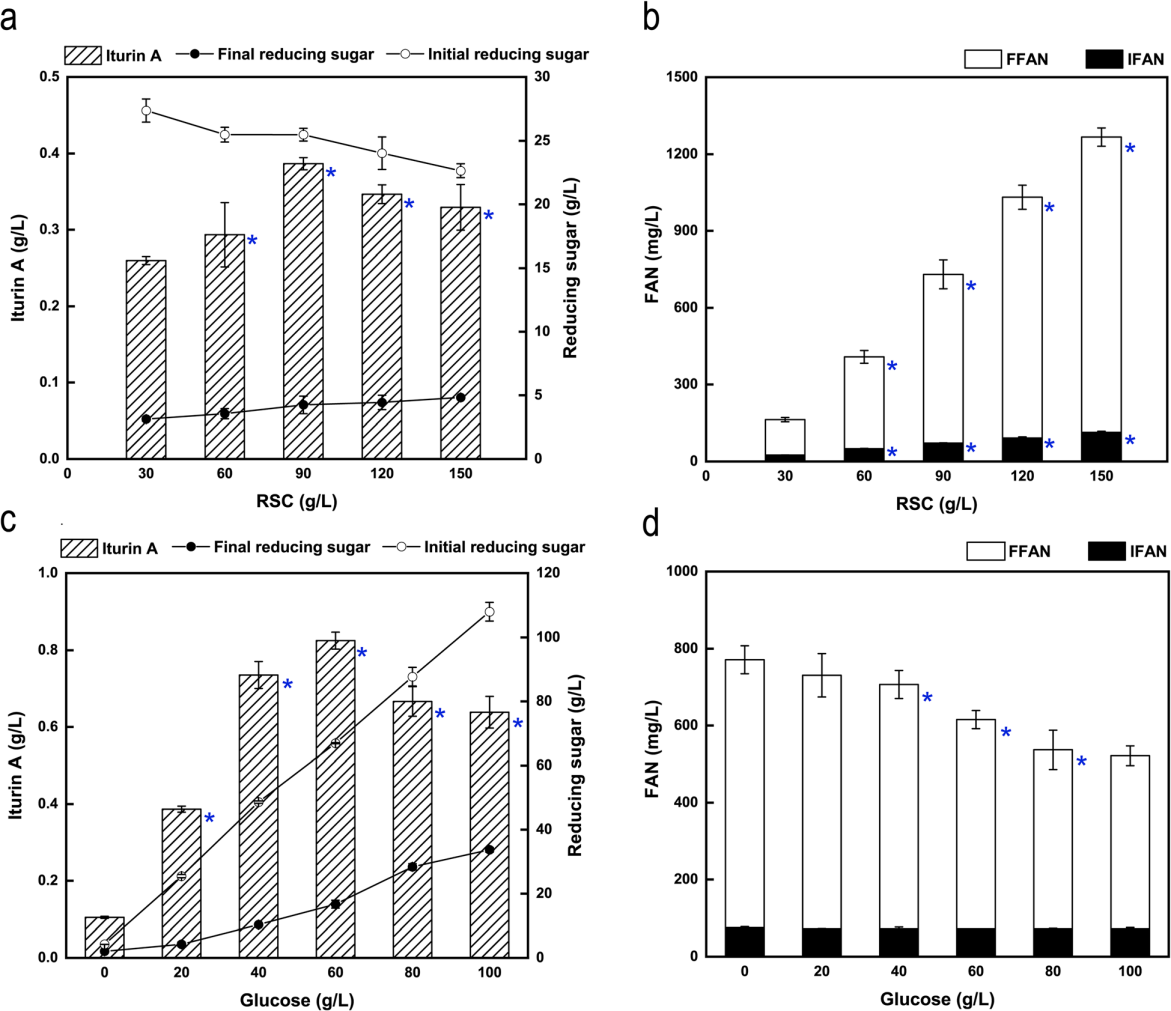
Effects of different initial RSC and glucose concentrations on iturin A production, concentrations of reducing sugars and FAN at 72 h in shake flasks. **a** Effects of different initial RSC concentrations on iturin A production, initial and final concentrations of reducing sugars. **b** Effects of different initial RSC concentrations on the concentrations of IFAN and FFAN. **c** Effects of different initial glucose concentrations on iturin A production, initial and final concentrations of reducing sugars. **d** Effects of different initial glucose concentrations on the concentrations of IFAN and FFAN. *P* < 0.05 was indicated by blue asterisk. In order to avoid the effects of fermentation volume among different flasks, the sample size was same of 1 mL from each flask for the analysis at the beginning and end of fermentation (0 and 72 h)

In our previous study [4], the optimal initial RSM and glucose concentrations for iturin A production by *B. subtilis* 3–10 in submerged fermentation were found to be 90 and 20 g/L, respectively. Therefore, 20 g/L initial glucose was used to test the influence of initial RSC concentration on iturin A production by *B. amyloliquefaciens* CX-20. As shown in Fig. 1a, the maximum iturin A final concentration 0.39 g/L was obtained when the initial RSC concentration was 90 g/L. With the increase of RSC concentration from 30 to 90 g/L, the production of iturin A increased. However, the iturin A production started to decrease upon further increase of RSC concentration from 90 to 150 g/L. An interesting phenomenon was that the final reducing sugar concentrations increased almost linearly with the initial RSC concentrations. This might be positively related to the reducing sugar released from RSC during fermentation process. The initial reducing sugar concentration slightly decreased, which was speculated to be related to the Maillard reaction caused by the high-temperature sterilization due to the covalent bonds formed between a free reactive -NH2 group of an amino acid and the carbonyl group of a reducing sugar [27]. The concentration of final free ammonium nitrogen (FFAN) also increased linearly from 162.50 to 1266.95 mg/L with the RSC concentration increasing from 30 to 150 g/L. Compared with the FFAN concentration, the initial free ammonium nitrogen (IFAN) concentration increased slightly (Fig. 1b). This indicated that *B. amyloliquefaciens* CX-20 had a strong ability to hydrolyze the insoluble nitrogen source in RSC to produce soluble form, which was not surprisingly considering the strong intrinsic protease activity of many *Bacillus* [4]. Genome and transcriptome analysis of *B. amyloliquefaciens* CX-20 demonstrated that it could not only produce proteases that hydrolyze proteins into peptides and amino acids, but also phytase, xylanase, cellulase and lipase enzyme, which was similar to *Aspergillus oryzae* and could result in the release of phosphate and the production of simple sugars to be used as carbon source for the growth of the microorganisms [28]. Therefore, *Bacillus* has intrinsic advantages for direct bio-utilization of RSC for the production of microbial metabolites [4, 5].

According to above results, the initial RSC concentration of 90 g/L was fixed to determine the optimum concentration of glucose and further improve the production of iturin A. Subsequently, the effects of different glucose concentrations ranging from 0 to 100 g/L on iturin A production were explored. It was clearly demonstrated that the optimal initial glucose concentration for iturin A production was 60 g/L, and the corresponding maximum iturin A concentration reached 0.82 g/L. Nevertheless, 0.10 g/L of iturin A was still produced even without adding glucose (Fig. 1c). As a complex mixture, RSM can not only be used as a nitrogen source [4, 5, 15, 17, 29], but can also provide carbon [14, 16] for the growth and metabolism of microorganisms. According to our results, RSC could also be used as both a carbon source and a nitrogen source. As a carbon source, RSC seemed to be efficient, since the final concentration of reducing sugars was 2.05 g/L from initial 4.22 g/L after 72 h fermentation. This further proved that *B. amyloliquefaciens* CX-20 could produce many enzymes to hydrolyze the carbohydrates of RSC into reducing sugars. It has been reported that rapeseed oil could be used as a source of carbon to ferment microbial products such as lipase [23], erythromycin [30] and isocitric acid [31]. However, whether the residual oil in RSC could be used as a carbon source for iturin A production by *B. amyloliquefaciens* CX-20 was still unclear. But RSC was insufficient to support the fermentation and synthesis of iturin A by *B. amyloliquefaciens* CX-20 as the sole carbon source. Accordingly, with the increase of initial glucose concentration from 0 to 60 g/L, the production of iturin A continued rising, but decreased when the initial glucose concentration was raised above 60 g/L. Although the final concentration of reducing sugars increased with the increase of initial glucose concentration, the change trend of the FFAN concentration (decreased from 771 to 522 mg/L) was opposite (Fig. 1d). This indicated that higher glucose or RSC concentrations could mutually promote the corresponding substrate consumption, but might not be necessary for improving iturin A production.

### Influence of lipase loading on the production of iturin A from RSC

**Fig. 2:**
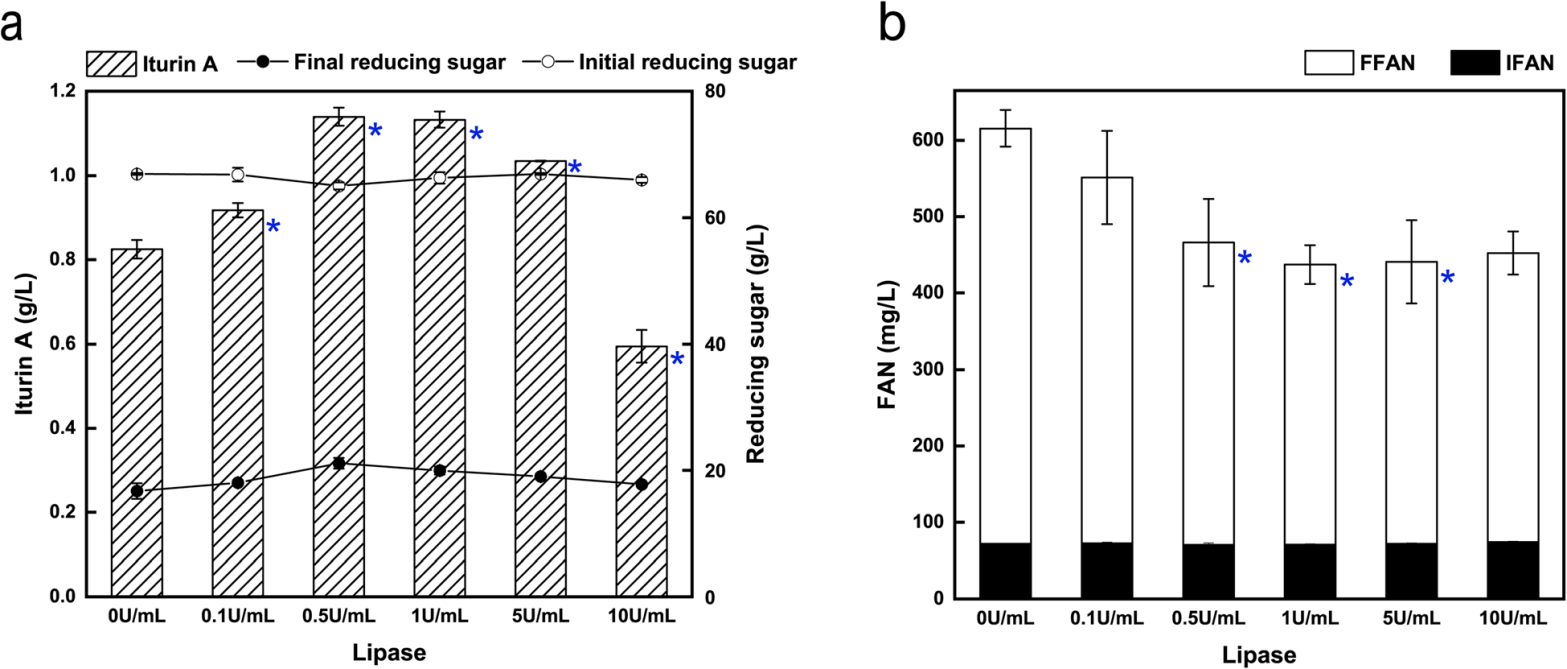
Effects of lipase loading on iturin A production, concentrations of reducing sugars and FAN at 72 h in shake flasks. **a** Effects of lipase loading on iturin A production, initial and final concentrations of reducing sugars. **b** Effects of lipase loading on the concentrations of IFAN and FFAN. *P* < 0.05 was indicated by blue asterisk. In order to avoid the effects of fermentation volume among different flasks, the sample size was same of 1 mL from each flask for the analysis at the beginning and end of fermentation (0 and 72 h)

Different concentrations of lipase (ranging from 0 to 10 U/mL) were added into the medium containing the optimal concentrations of 90 g/L RSC and 60 g/L glucose at the beginning of the process to enable simultaneous hydrolysis and fermentation. As shown in Fig. 2, with the increase of lipase concentration from 0 to 0.5 U/mL, the production of iturin A gradually increased. However, with the further increase of lipase concentration (from 0.5–10 U/mL), the production of iturin A began to decrease. When the concentration of lipase was 0.5 U/mL, the iturin A production reached a maximum of 1.14 g/L, which represented a 38.15% increase over the fermentation without any lipase addition. By contrast, when the concentration of lipase reached 10 U/mL, the final concentration of iturin A decreased to 0.59 g/L, which was even 27.94% lower than that without any lipase addition. The change trend of the final reducing sugar concentration was similar to that of iturin A production. Lipases are a group of enzymes that hydrolyze the ester bonds in triacylglycerides to form fatty acids and glycerol, or catalyze the synthesis of esters under certain conditions [32]. The lipase used in this study was a mixture used as a feed additive to aid animal digestion. Therefore, we speculated that the appropriate addition of lipase might help hydrolyze the residual oil in RSC into fatty acids and glycerol. Notably, glycerol has been proved to be a more suitable carbon source for iturin A production than glucose (data not shown). This helps explain why the FFAN concentrations with added lipase was lower than that without lipase, since the released glycerol could also promote the consumption of the nitrogen source (Fig. 2b). However, excess lipase had a negative effect on the production of iturin A.

### Influence of lipids and lipase on the fermentation of iturin A

**Fig. 3:**
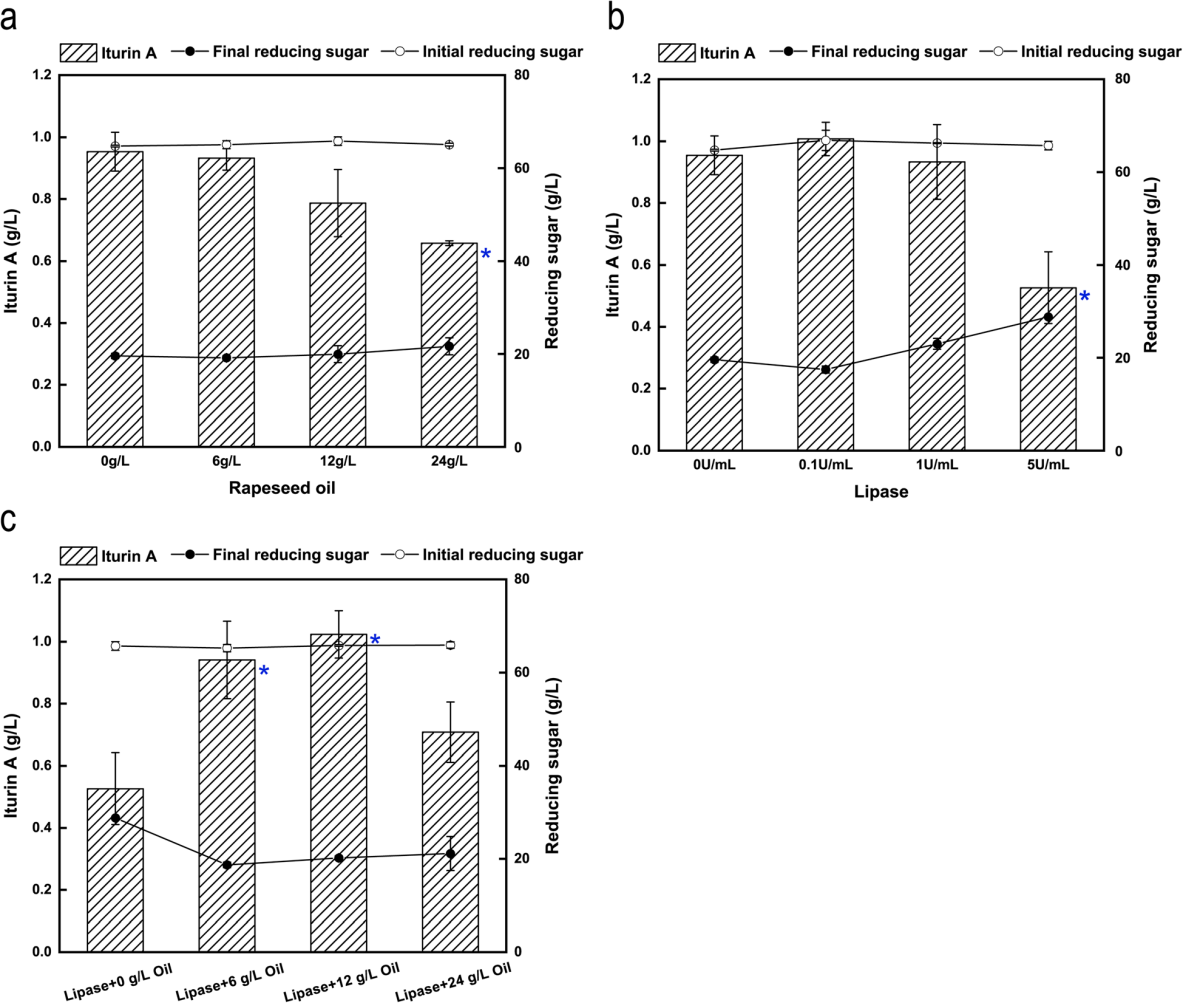
Effects of different rapeseed oil and lipase concentrations. **a** Effects of different rapeseed oil concentrations on iturin A production, initial and final concentrations of reducing sugars. **b** Effects of different initial lipase concentrations on iturin A production, initial and final concentrations of reducing sugars. **c** Effects of different ratio of lipase and rapeseed oil on iturin A production, initial and final concentrations of reducing sugars. *P* < 0.05 was indicated by blue asterisk. In order to avoid the effects of fermentation volume among different flasks, the sample size was same of 1 mL from each flask for the analysis at the beginning and end of fermentation (0 and 72 h)

As shown in Table 1, the content of protein in RSC was 33.5% while the protein content in RSM was 39.4%. Therefore, the protein content in 90 g/L RSC was equal to that of 76.52 g/L RSM, and the latter was added to substitute 90 g/L RSC to simulate the protein content in the original medium. Because the crude fat content of RSM was 1.6% while that of RSC was 14.4%, the crude fat content of the medium containing 90 g/L RSC was 12.96 g/L, while that of the medium containing 76.52 g/L RSM was 1.22 g/L. The difference of crude fat between the two media was 11.74 g/L. In order to explore the effect of crude fat on iturin A production, 0, 6, 12 or 24 g/L of natural rapeseed oil was added into the medium containing 76.52 g/L RSM (Fig. 3a). After 72 h of fermentation, the final concentration of iturin A without added oil was 0.95 g/L, which was 15.56% higher than that of the 90 g/L RSC medium. With the increase of oil concentration, the trend of iturin A production was gradually decreasing. When 12 g/L rapeseed oil was added into the medium, the iturin A production decreased to 0.79 g/L. Although the production of iturin A decreased 17.46% compared to that produced without any oil addition, its value was very close to that produced with 90 g/L RSC (0.82 g/L). When 6 g/L rapeseed oil was added to the medium, the iturin A production decreased to 0.93 g/L, which was very close to the value obtained without adding oil. Because the lipopeptide products possess surfactin activity and cause foam formation, it is difficult to control the fermentation, which also restricts the industrialization of lipopeptide production [2]. Rapeseed oil has been found to be an efficient antifoam compound [33]. Our results also demonstrated that when the concentration of added rapeseed oil was lower than 0.6%, there was almost no negative effect on iturin A production. Therefore, rapeseed oil seemed to also be a suitable antifoam compound for iturin A production. However, when 24 g/L rapeseed oil was added into the medium, the iturin A production decreased to 0.66 g/L.

From the results shown in Fig. 2, we found that an appropriate concentration of lipase could improve the production of iturin A. Conversely, its production would be reduced when the concentration of lipase was too high. The oil content of RSM was lower than that of RSC (Table 1). Therefore, in theory, the optimal concentration of lipase for RSM should decrease accordingly. As expected, when the concentration of lipase was 0.1 U/mL, the iturin A production had a slight increase, from 0.95 g/L to 1.01 g/L, but when the lipase concentration was increased to 1 U/mL, the final concentration of iturin A was slight lower than that without any lipase added (0.95 g/L vs. 0.93 g/L). When the lipase concentration was increased to 5 U/mL, the final concentration of iturin A drastically decreased to 0.53 g/L, which was only 55.14% of that produced without any added lipase (Fig. 3b). At the same time, the final reducing sugar concentration gradually increased with the increase of lipase concentration when the lipase concentration was more than 0.1 U/mL.

Although the production of iturin A decreased slightly when RSC was used as nitrogen source if the lipase concentration was increased to 5 U/mL, the production of iturin A apparently decreased when RSM containing an equal protein content was used as the nitrogen source. Therefore, proper proportions of rapeseed oil and lipase appeared to be crucial for optimal iturin A production. As shown in Fig. 3b, a lipase concentration of 5 U/mL had a significant influence on the production of iturin A from RSM, and was chosen to explore the appropriate ratio of rapeseed oil to lipase. As shown in Fig. 3c, with the increase of rapeseed oil concentration from 0 to 12 g/L, iturin A production continued rising and reached a maximum of 1.02 g/L, which was almost equal to the iturin A production (1.03 g/L) produced from 90 g/L RSC with 5 U/mL lipase, when the addition of rapeseed oil was 12 g/L. However, when the concentration of rapeseed oil was increased to 24 g/L, the final concentration of iturin A decreased to 0.61 g/L, which was only 59.42% of that obtained with 12 g/L rapeseed oil.

**Fig. 4:**
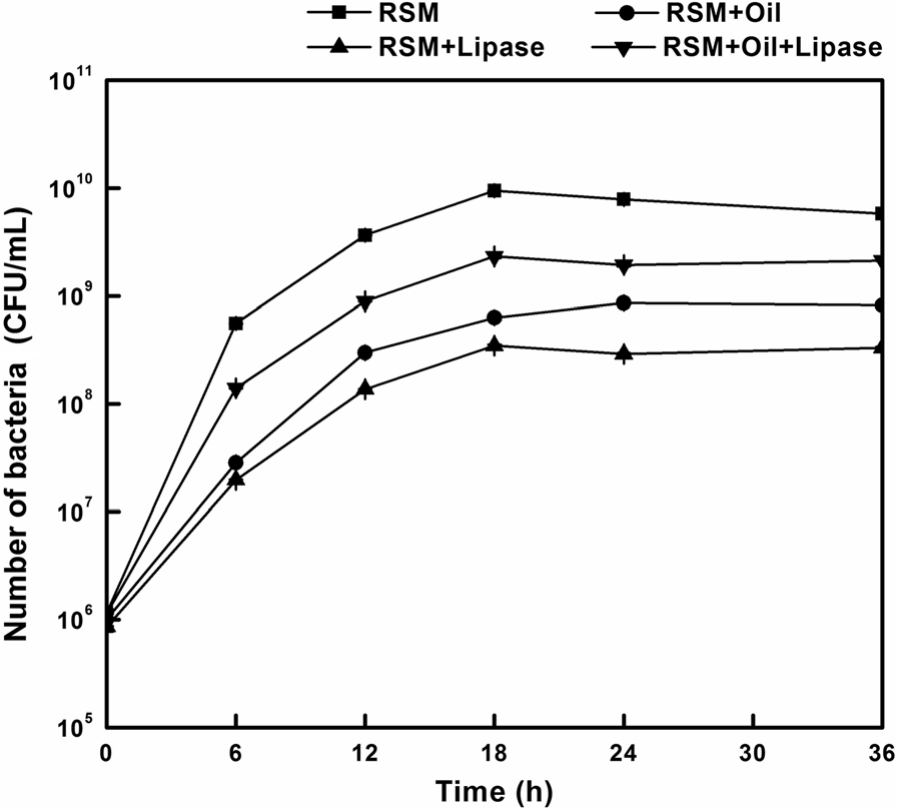
Effects of 12 g/L rapeseed oil and 5 U/mL lipase on the growth of *Bacillus amyloliquefaciens*. In order to avoid the effects of fermentation volume among different flasks, the sample size was same of 0.5 mL from each flask for the analysis at 0, 6, 12, 18, 24 and 36 h, respectively

There are many possible explanations why lipase and rapeseed oil could reduce each other’s negative effects on iturin A production. The most likely one is related to microbial growth. Therefore, we examined the growth curves of *Bacillus* under several representative conditions. As shown in Fig. 4, when 12 g/L rapeseed oil or 5 U/mL lipase was added separately into the medium containing 76.52 g/L RSM, both the specific growth rates (0.56 and 0.52 h^− 1^ from 1.03 h^− 1^) and the ultimate maximum viable cell count (about 8 × 10^8^ and 3 × 10^8^ mL^− 1^ from about 8 × 10^9^ mL^− 1^) were significantly reduced compared with the medium without either rapeseed oil or lipase. However, when 12 g/L rapeseed oil and 5 U/mL lipase were added at the same time, both the specific growth rate (0.80 h^− 1^) and the ultimate maximum viable cell count (about 2 × 10^9^ mL^− 1^) showed an obvious recovery.

## Discussion

The feasibility of using RSM as nitrogen sources in microbial fermentation processes has been reported [4, 5, 14–18]. However, as the low-cost by-product of the processing of oil crops, RSC also contains a high content of oil in addition to its protein content. Whether the residual oil in RSC might affect the production of microbial metabolites including iturin A, and whether lipase could be useful for improving their productions, were still not clear.

In this study, the feasibility of using untreated RSC as nitrogen source to produce the valuable cyclic lipopeptide antibiotic iturin A using *B. amyloliquefaciens* CX-20 in submerged fermentation was first investigated. The maximum production of iturin A was 0.82 g/L at the optimal initial RSC and glucose concentrations of 90 and 60 g/L, respectively. However, when RSC was substituted with RSM as nitrogen source based on equal protein content, the final concentration of iturin A was improved to 0.95 g/L. Excess rapeseed oil over 6 g/L would suppress the production of iturin A in *B. amyloliquefaciens* CX-20. Kamzolova et al. [31] used rapeseed oil as a source of carbon and energy to produce isocitric acid in the unconventional yeast *Yarrowia lipolytica*. The first step of utilization of rapeseed oil by the yeast was its hydrolysis by extracellular lipases that produce glycerol and fatty acids. Moreover, the fatty acid profile of rapeseed oil (%, by mass) was found to be C16:0, 4.0; C18:0, 1.2; C18:1, 58.8; C18:2, 28.1; C18:3, 5.9 with a total unsaturated fatty acid mass fraction of 93.6% [34]. Therein, palmitic acid was proved to be a useful precursor whose addition could enhance the production of iturin A [19]. Oils are the essential components of industrial fermentation media and have been routinely added to media for the production of secondary metabolites. They have been used as antifoams, sole carbon sources, auxiliary carbon sources, to provide precursors for antibiotic synthesis and to remove the antibiotic from bacterial access and reduce its suppressive effect on antibiotic production [30]. However, it was considered possible that oil might form a thin film on the surface of the medium, decreasing the oxygen dissolution efficiency, which may influence the production of iturin A [35]. Wu et al. [19] verified the negative effects on iturin A production after the addition of either palm or soybean oil. Our results confirmed that rapeseed oil also had a negative effect on iturin A production, which might be a key factor influencing the utilization of RSC.

The nutrients in rapeseed oil by-products cannot be directly assimilated by the majority of industrial microorganisms without pretreatment due to its particular physical and chemical structure [27, 36]. Although many studies investigated ways of using enzymes such as proteases, cellulase and viscozyme to enhance the value of nitrogen from the rapeseed by-products for microbial fermentations [16, 29], we are not aware of any studies on utilizing lipase to improve the substrates’ value for microbial production. To our best knowledge, this might be the first study on directly using lipase for the production of iturin A from rapeseed oil by-products. At the optimal lipase concentration of 0.5 U/mL, the maximal production of iturin A from RSC reached 1.14 g/L, which was 38.15% higher than that without any lipase supplement. The experiments of RSC substitution with RSM based on equal protein content further proved that excess concentration of rapeseed oil or lipase would have a negative effect on iturin A production. However, the proper ratio of these two elements for simultaneous fermentation by *B. amyloliquefaciens* CX-20 would mitigate the effects of each, and could even boost the amount of iturin A produced from RSC. These variation trends seemed similar to that of microbial growth. Therefore, we concluded that the negative effect caused by rapeseed oil or lipase might be related to their corresponding effects on cell growth, although this was likely not the only reason. After all, when both were added at the same time, although the growth rate and the ultimate maximum viable count were still lower than that of the control, the production of iturin A was slightly higher. This was consistent with the study by Jin et al. [4] whose research demonstrated that ammonium nitrate was a good nitrogen source for *Bacillus* growth while it was not suitable for iturin A production. More work would be needed to further elucidate this phenomenon.

**Fig. 5:**
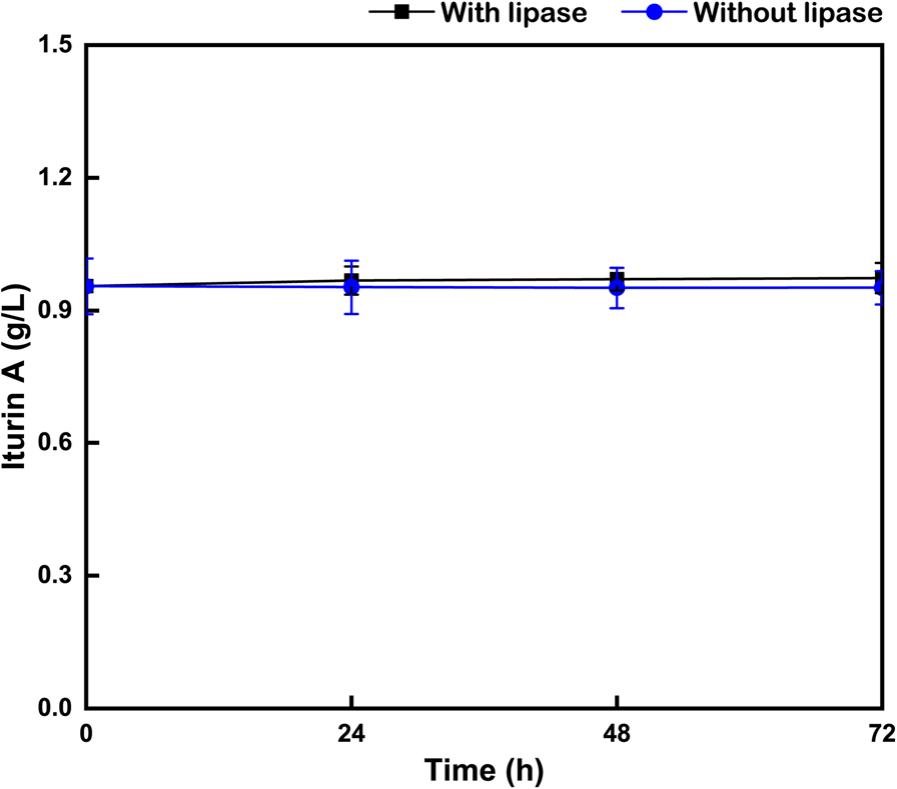
Effects of excess lipase on the stability of iturin A. In order to avoid the effects of fermentation volume among different flasks, the sample size was same of 0.5 mL from each flask for the analysis at 0, 24, 48 and 72 h, respectively

Iturin A consisted of a cyclic heptapeptide linked to a 14–17 carbons β-hydroxy fatty-acid chain. Therefore, it was speculated that lipase might cleave the fatty acid chain of iturin A so that reduced iturin A production. However, as shown in Fig. 5, when the deviations were taken into consideration, the addition of excess lipase (5 U/mL) after 72 h fermentation had almost no effect on the stability of iturin A from RSM, compared with that without any lipase supplement. Although lipopeptides have been reported to have excellent thermal and chemical stability [2], this might be the first study to investigate the good resistance of iturin A to lipase.

## Conclusion

Compared with RSM, RSC has been rarely used as a raw material for microbial fermentation due to its high content of residual oil. This study not only demonstrated that the residual oil in RSC had a negative effect on iturin A production, but also provided an efficient means to solve this problem by adding a commercially-available feed-processing lipase for simultaneous hydrolysis and fermentation. When the optimal lipase concentration of 0.5 U/mL was added into the RSC medium, the final concentration of iturin A increased from 0.82 to 1.14 g/L, which was also higher than the 0.95 g/L produced in RSM medium containing an equal protein content. By using RSM and rapeseed oil to simulate RSC, excess rapeseed oil was proved to suppress the production of iturin A, which might be related to the oil forming a thin film on the surface of the medium, and thus decreasing the oxygen dissolution efficiency and cell growth. The proper supplementation ratio of the lipase for simultaneous hydrolysis and fermentation by *B. amyloliquefaciens* CX-20 was verified to mitigate the effect of rapeseed oil for the first time in this study. This research is important not only for improving the economic value of RSC and decreasing the production cost of iturin A, but also for establishing a new way to increase the value of rapeseed oil for microbial fermentation, either as antifoams, auxiliary carbon sources, or providing precursors for antibiotic synthesis.

## Data Availability

All data generated or analysed during this study are included in this published article.

## Abbreviations

CFU: Colony forming units
FAN: Free ammonium nitrogen
FFAN: Final free ammonium nitrogen
IFAN: Initial free ammonium nitrogen
LB: Luria-Bertani
RSC: Rapeseed cake
RSM: Rapeseed meal
